# The New Media Landscape and Its Effects on Skin Cancer Diagnostics, Prognostics, and Prevention: Scoping Review

**DOI:** 10.2196/53373

**Published:** 2024-04-08

**Authors:** Priscilla L Haff, Alli Jacobson, Madison M Taylor, Hayden P Schandua, David P Farris, Hung Q Doan, Kelly C Nelson

**Affiliations:** 1 McGovern Medical School The University of Texas Health Science Center at Houston Houston, TX United States; 2 Cancer Prevention Research Training Program Houston, TX United States; 3 Davidson College Davidson, NC United States; 4 University of Texas MD Anderson Cancer Center Houston, TX United States

**Keywords:** social media, communication, skin cancer, melanoma, misinformation, scoping review

## Abstract

**Background:**

The wide availability of web-based sources, including social media (SM), has supported rapid, widespread dissemination of health information. This dissemination can be an asset during public health emergencies; however, it can also present challenges when the information is inaccurate or ill-informed. Of interest, many SM sources discuss cancer, specifically cutaneous melanoma and keratinocyte cancers (basal cell and squamous cell carcinoma).

**Objective:**

Through a comprehensive and scoping review of the literature, this study aims to gain an actionable perspective of the state of SM information regarding skin cancer diagnostics, prognostics, and prevention.

**Methods:**

We performed a scoping literature review to establish the relationship between SM and skin cancer. A literature search was conducted across MEDLINE, Embase, Cochrane Library, Web of Science, and Scopus from January 2000 to June 2023. The included studies discussed SM and its relationship to and effect on skin cancer.

**Results:**

Through the search, 1009 abstracts were initially identified, 188 received full-text review, and 112 met inclusion criteria. The included studies were divided into 7 groupings based on a publication’s primary objective: misinformation (n=40, 36%), prevention campaign (n=19, 17%), engagement (n=16, 14%), research (n=12, 11%), education (n=11, 10%), demographics (n=10, 9%), and patient support (n=4, 3%), which were the most common identified themes.

**Conclusions:**

Through this review, we gained a better understanding of the SM environment addressing skin cancer information, and we gained insight into the best practices by which SM could be used to positively influence the health care information ecosystem.

## Introduction

As of April 2023, 4.8 billion people, or 59.9% of the world’s population, were identified as social media (SM) users [1]. In the age of omnipresent internet exposure, more people than ever receive and seek medical information from SM. More than 80% of US state health departments have an SM account, and SM has become a safe space for patients with cancer to discuss diagnoses and seek education [2]. Over 80% of patients with cancer reported using SM to connect with peers, and over 77% of patients with cancer cited the internet as the most important source of medical information [3]. When compared to legacy public health forums, SM and the new media landscape carry both promise and risk. While accurate information can be rapidly distributed, so can misinformation, and this spread happens at a pace and scale that is inconceivable to prior communication environments [4].

Our scoping review focuses specifically on SM information and skin cancer, including melanoma and keratinocyte cancer (basal cell and squamous cell carcinoma). While keratinocyte cancers are more common, melanoma carries a higher risk of mortality [5] and is projected to be the second most common cancer in the United States by 2040 [6]. Melanoma offers opportunities for primary, secondary, and tertiary prevention. Campaigns for ultraviolet exposure reduction, skin cancer risk factors education, and guideline-concordant care awareness are all uniquely positioned for SM-based efforts. In this review, we explore how SM interfaces with skin cancer information and dissect the current research landscape as it pertains to this topic.

## Methods

### Overview

Scoping reviews are exploratory studies that aim to examine the extent of research performed on a given topic [7]. While similar to systematic reviews, scoping reviews differ in that they are broad and do not synthesize data via a meta-analysis. Scoping reviews are useful because they provide an organized description of the available literature, particularly with topics that have been heavily studied from various perspectives [8].

### Search Strategy

A medical research librarian (DPF) developed a systematic search for relevant papers in MEDLINE, Embase, Cochrane Library, Web of Science, and Scopus covering January 1, 2000, to June 9, 2023. Publications were not limited by geography. The search was limited to texts that had full-text availability in the English language and discussion of the new communication environments and skin cancer. The search used controlled vocabulary and language terms selected to include SM and skin cancer. Search sensitivity was tested by the ability of preliminary search strategies to include known, relevant citations. The full search strategy can be found in Multimedia Appendix 1.

### Eligibility Criteria

The inclusion and exclusion criteria are listed in Textbox 1. Studies that were eligible for inclusion investigated the connection between skin cancer and SM. The search was conducted between January 1, 2000, and June 9, 2023, to limit the number of papers and to only include records that were relevant to this era of new communication, after the SM boom.

**Inclusion and exclusion criteria.**

**Inclusion criteria**

- Melanoma
- Keratinocyte cancer (Basal cell carcinoma, Squamous cell carcinoma)
- X (Twitter)
- Facebook
- Instagram
- TikTok
- YouTube
- Pinterest
- Other forms of new media
- Tanning ideation
- Skin cancer prevention

**Exclusion criteria**

- Conference abstracts
- No full-text availability
- No translation to English language
- Unfinished study
- Artificial intelligence technology rather than social media
- Teledermatology rather than social media
- Not dermatologic information
- No skin cancer information
- No social media information

### Data Extraction

Two authors (PLH and AJ) independently screened the titles and abstracts of each citation produced by the search strategy using the inclusion and exclusion criteria to decide which papers would progress to full-text review. Each record was reviewed twice, and, if a conflict was found, the lead investigator (KCN) would make the final decision. The full texts of all potentially eligible records were then analyzed independently by the investigators. Disagreements were resolved by reexamination and discussion. A flowchart was developed using the PRISMA (Preferred Reporting Items for Systematic Reviews and Meta-Analyses) reporting guidelines to demonstrate the study selection process (Multimedia Appendix 2) [9]. Author, publication year, study type, geographic location, platform investigated, principal findings, and STROBE (Strengthening the Reporting of Observational Studies in Epidemiology) score were extracted from each included publication. A copy of the STROBE score criteria can be found in Multimedia Appendix 3 [10]. The STROBE scoring system was used to ensure this review included high-quality studies.

The included publications were divided into 7 categories based on the primary evaluated aspect of the study: engagement, campaigns, demographics, research, education, patient support, and misinformation. To be included in the engagement category, a publication must discuss an attribute of interaction, participation, connection, and involvement designed to illicit a result [11]. Engagement can be understood as the likes, comments, and shares posts acquire. Campaigns include publications that describe a new media intervention designed to promote primary or secondary skin cancer prevention and its effect on the population. A publication was included in the demographics category if it discussed demographic differences in skin cancer SM advertising. The research category encompasses papers that demonstrate how SM aids in skin cancer research recruitment. A publication in the education category must discuss a way new media communication can be used for physician-to-physician or physician-to-patient skin cancer education. The patient support category includes records that demonstrate how the new communication environment lends itself to supporting patients with skin cancer. Scientific misinformation is defined as misleading information relative to the best available scientific evidence [12]. Therefore, to be included in the misinformation section, a publication must discuss false information dissemination or poor information quality regarding skin cancer across SM platforms.

## Results

### Overview

**Figure 1:**
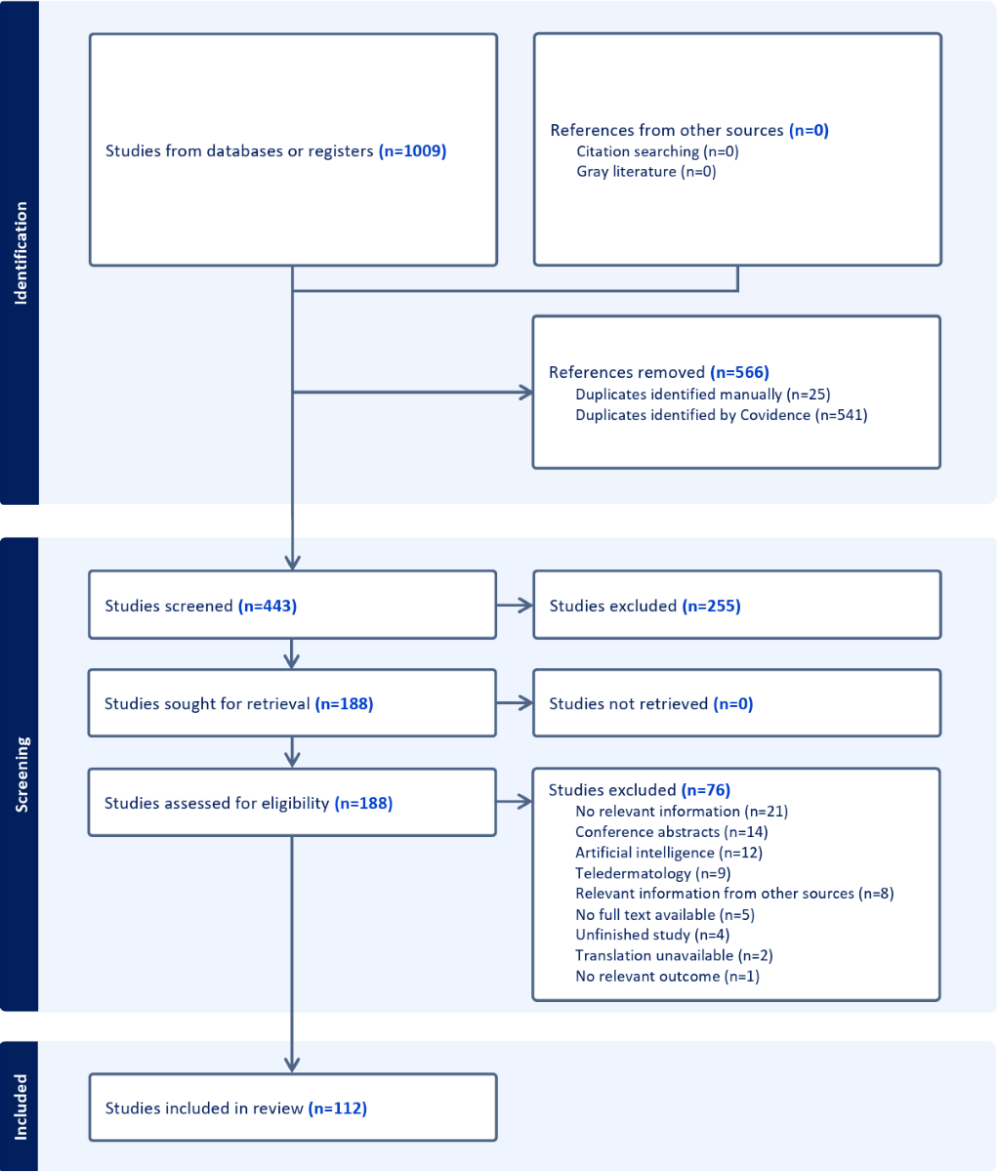
PRISMA (Preferred Reporting Items for Systematic Reviews and Meta-Analyses) flow diagram for study inclusion.

We identified 1009 records through the initial search, with the removal of 556 duplicate records via Covidence (Veritas Health Innovation; Figure 1). Two investigators (PLH and AJ) independently screened the remaining studies’ titles and abstracts, with 188 records receiving full-text review. After full-text review, 76 were excluded through dual reviewer evaluation. Records with contradictory decisions were sent to a third-party reviewer (KCN), who provided the deciding vote. The included studies were divided into 7 groupings based on the publication’s primary objective: misinformation (n=40, 36%), prevention campaign (n=19, 17%), engagement (n=16, 14%), research (n=12, 11%), education (n=11, 10%), demographics (n=10, 9%), and patient support (n=4, 3%), which were the most common identified themes. The data were extracted from each record into a characteristics table (Multimedia Appendix 4 [5,13-123]).

### Engagement

X (previously known as Twitter) has enormous potential for public health engagement; of the 112 included papers, 16 were included in the category of engagement [13]. X is more public than Instagram or Facebook and is used more often than other SM platforms to promote scientific papers and increase interactions with scientific literature [14]. On X, the top hashtag for skin cancer is #melanoma, and the key drivers of discussion are patient-focused entities [15]. Posts using shock or humor generate the most likes or comments, and informative posts are most likely to be shared [16]. Engagement with posts about skin cancer correlates not with skin cancer incidence in a given geography, but instead with SM literacy of the exposed users [17]. To optimize the impact of X as a tool for skin cancer engagement, more information is needed to increase message dissemination and uniformity [18].

TikTok is a rapidly growing new media platform with over 755 million users in 2022 [124]. The most popular skin cancer content on TikTok includes videos with on-screen text and health care attire, such as a white coat or scrubs [19]. Skin cancer is among the top 8 dermatological TikTok topics, with patient testimonies being the most common format, followed by educational videos and clinical demonstrations [20].

Most Instagram content addressing skin cancer originates from influencers and celebrities, not dermatologists [21]. Instagram offers a venue for patients to share their skin cancer journey (often with the #skincancerawareness hashtag [22]) and increase users’ exposure to skin cancer information. Instagram posts referencing negative emotions (fear and anger), physical consequences, technical treatment information, or real skin cancer images increase audience interactivity, while positive posts have no effect on engagement [23].

This trend continues with Facebook, where the most-used technique to increase audience engagement is inducing fear [24]. Like X, Facebook posts with a humorous element increase viewer satisfaction and attention [25]. One advertising study compared Facebook user engagement of a parody video, a celebrity video, or a fact-based video regarding skin cancer and found engagement to be the highest for the parody video [25]. Facebook also allows individuals to post their personal skin cancer narratives. For example, Tawny Willoughby went viral due to a graphic selfie of her significant facial inflammation during treatment with topical 5% 5-fluorouracil: the post received over 50,000 views and was correlated to a 162% increase in internet search queries about skin cancer [26].

Increased user interactivity correlates with enhanced engagement with the information. This trend is consistent across platforms but is specifically noticed in support groups and on websites. Support groups are particularly effective if they are larger and have active, web-based comment sections [27], whereas the interactivity of skin cancer websites promotes an individual’s intention to use sun protection [28].

### Prevention Campaigns

The category of prevention campaigns encompassed 19 of 112 included papers. The YouTube video “Dear 16-year-old Me” is a prime example of a successful SM prevention campaign. This video uses mixed emotion methods to address the importance of sun protection, which amplifies the impact of the message by evoking compassion to increase positive social behaviors [29,30]. After viewing the video, surveys demonstrated increased viewer intent to pursue a professional skin examination [31]. The video made a compounding impact when presented alongside lighthearted face-aging software [32].

Other YouTube skin cancer awareness campaigns include the “It’s a beautiful day ... for Cancer” and “Don’t be a Lobster.” The “It’s a beautiful day ... for Cancer” video was an ironic music video that spurred conversation of sun protection behaviors: it received 250,000 views, and 44% of viewers reported changed opinions on sun protection [31]. The “Don’t be a Lobster” campaign consisted of an anonymous YouTube video highlighting the replacement of the red dragon of the Welsh flag with a red lobster. This anonymity and clever placement of the red lobster image quickly gained media attention and started the viral campaign. The campaign’s effectiveness was quantified by Google Trends, showing a 10% increase in skin cancer and a 300% increase in “sun cream” searches [33].

X’s #dontfryday made a significant impact globally, with over 12 million impressions. The most influential posts were sent out by celebrities. One study found that while noncelebrity individuals contributed the most content for the campaign, celebrities made a monumental impact, with only 18 contributors generating 8,735,549 impressions [34,35].

As seen with #dontfryday, celebrity influence plays a huge role in enhancing the success of a prevention campaign. Actor Hugh Jackman has posted his skin cancer experience on SM. Each time he posts, the search “skin cancer” spikes on Google [36,37]. Like Jackman, Dayanara Torres, a former Miss Universe, used her platform to discuss her diagnosis of melanoma. One dermatology clinic in New Jersey noted that after Torres’ announcement, many Hispanic patients came to their clinic specifically with skin cancer screening concerns rather than their usual motivating factors [38]. Now, Torres partners with the Melanoma Research Foundation as a spokesperson for the #GetNaked awareness campaign, promoting monthly self-screenings and yearly dermatologist skin examinations [125]. In Portugal, athletes distributed skin cancer screening messages, and by the end of the study, more individuals were screened than in the previous years [39].

SM can perpetuate the tanned ideology, but with targeted interventions, this risk can be mitigated. Appearance-focused interventions, or interventions that use aging, wrinkles, and sunspots in their educational material, successfully reduced Instagram users’ positive associations with SM images featuring people with tanned skin [40]. Increasing SM literacy can also decrease the internalization of the tanned ideology. SM literacy is the ability of a user to evaluate and critically analyze posts, which aims to promote greater skepticism of appearance-related media [41,42]. The self-persuasion theory is another method that can predict healthy behaviors and enhance skin protection intentions: individuals who share skin protection information predictably use those same practices [43-45].

A Danish antisunbed campaign focused on decreasing tanning bed use among adolescents, generating intense public debate, and increasing legislative support [46]. With the new legislation, a parent must sign off on indoor tanning if a child is younger than 18 years. Targeting educational messages to mothers is a promising approach, as mothers who are more educated about the dangers of indoor tanning and equipped to discuss those dangers are less likely to allow their children to use tanning beds [47].

### Demographics

In total, 10 of the 112 papers were categorized in the demographics group. The new communication environment offers an opportunity for skin cancer prevention but primarily targets younger demographics: the success of SM skin cancer prevention campaigns decreases as participant age increases [48-50]. However, many young adults consider SM prevention messages to be uninfluential, because they are lost in the influx of other information [51,52].

One underrepresented demographic is individuals with darker-pigmented skin, as many skin cancer educational and prevention messages do not engage these populations. For example, 97% of skin cancer pins on Pinterest were of white skin individuals [53]. Similarly, a review demonstrated that 100% of skin cancers depicted on SM advertisements had a background of Fitzpatrick type I or II skin [54]. SM representation is critical, as a study that interviewed 27 African American individuals found SM to be a primary means by which people with darker pigmentation are exposed to public health messages related to skin cancer [55]. Participants also stated it would be important for skin cancer awareness messages on SM to feature Black communities to feel that the information is relevant to them [55].

Sexual orientation and gender identification also have a role in engagement and prevention advertising [56]. Indoor tanning motivations in sexual minority men have not been investigated; thus, targeted prevention campaigns are lacking. Compounding, sexual minority men are specifically targeted by tanning salons through SM marketing, further encouraging deleterious tanning behaviors in this population [57].

### Research Recruitment

In total, 12 of the included 112 papers were designated as research recruitment, collecting a total of 2912 patient responses [5,58-63]. By distributing surveys through SM platforms, scientists can recruit patients with rare skin cancers (such as dermatofibroma sarcoma protuberans [58]) and distribute research recruitment efforts globally. Additionally, SM can be used in studies to assess patients’ health-related quality of life. This concept was validated in one such study, which showed the alignment of current electronic health record data to SM data mining of symptoms that are common for patients receiving skin cancer treatment [64,65]. SM can also support data crowdsourcing to help physicians understand the patient experience and identify high-risk individuals for prevention [66,67]. New communication technology offers a unique opportunity for physicians to directly communicate with and understand their patients on a deeper level [68].

### Education

Education through new media resources allows dermatologists to have a more substantial global reach in skin cancer prevention, which is what was primarily discussed in the 11 papers included within this category. In the past, studies have shown that the presence of dermatology-related content from reputable journals on SM is limited [69-72]. It is effective to use social networking sites to provide an avenue for health care providers to communicate, share knowledge, and discuss care [73]. For example, Doximity is a platform for health professionals to freely discuss topics such as skin cancer. Dermatologists can use Doximity to share skin cancer awareness messages, prevention strategies, or scientific papers with the broader physician community. Anyone can then share information from Doximity to SM sites to reach the wider patient population [74].

Similarly, physicians share posts during the American Society of Clinical Oncology meeting. From 2011 to 2012, “melanoma” was a trending term at the American Society of Clinical Oncology conference, and attending physicians dispersed the latest scientific research over X [75]. Physicians can also connect with patients and teach proper skin self-examination through SM [76]. One study noted that 79% of patients had increased confidence in performing skin self-examination after watching eHealth YouTube videos, which proved superior to classic methods such as informational brochures [77].

Education strategies using beauty technicians can also serve as an intervention tactic for skin cancer. For example, the Pele Alerta Project built a website to assist beauty professionals in the early detection of skin cancers [78]; in addition, tattoo artists were targeted to provide skin protection information in their aftercare instructions [79]. Each educational opportunity gives patients a greater chance of catching their skin cancer early.

### Patient Support

In total, 4 of the 112 included papers discussed social media and its use in patient support. Patients often use SM to share their firsthand experiences, such as skin cancer excision procedures, to help provide realistic expectations for other patients [80]. They also use SM to discuss the effects of skin cancer on their quality of life. Mental health struggles and uncertainty were the 2 most common themes for forums for patients with skin cancer [81], and emotional burden, treatment, and diagnosis were common conversation topics throughout these support groups [82]. Over 52% of melanoma Facebook groups are used to support patients [83].

### Misinformation

Finally, the majority of included records discussed misinformation, with 40 of 112 papers belonging to this category. Participants in one study viewed a misinformation video and afterward had less intention to wear sunscreen, demonstrating the detrimental effect of misinformation. Comments posted correcting the misinformation in the video showed no significant increase in attitudes regarding sunscreen use [84].

Many misinformation studies verify a positive correlation between SM use and indoor tanning behaviors [85-87]. Not only does SM propagate skin tone dissatisfaction, but it also has provided a place of advertisement for tanning salons. Indoor tanning businesses propagate misleading information to increase their customer base, such as “indoor tanning is a safe way to get vitamin D” [88,89]. Companies have used “#paleshaming” to bring adolescents to their salons by damaging their self-esteem and motivating their engagement in tanning behaviors [90]. Not only do tanning salons use SM for business promotion, but also tanning, in general, is glorified across new media [91]. A review of tanning hashtags was conducted for TikTok, Pinterest, YouTube, and X, where 90%, 85%, 68%, and 68.9% of tanning content was positive, respectively [92-95]. Further research showed that, over a 2-week period, only 2.56% of 154,496 tanning posts on X mentioned skin cancer as a risk [96]. In summary, SM propagates indoor tanning behaviors by adding to skin tone dissatisfaction, advertising for tanning salons, and broadcasting a positive attitude toward tanning and sunburn.

YouTube attracts over 866 million users monthly [97]. Multiple studies identified that the current YouTube video landscape is of low quality, reliability, understandability, and actionability [98-107]. A table with the extrapolated results from each quality analysis study can be found in Multimedia Appendix 5 [98-107]. While there has been a positive progression in educational content on YouTube from 2014 to 2018 [108,109], misinformation and low-quality information still plague the viewing streams. For instance, YouTube creators grossly overestimate the relationship between COVID-19 and vitamin D, encouraging tanning behaviors during the pandemic [110]. Similarly, multiple studies found blatant misinformation from many YouTube videos regarding alternative therapies, especially concerning “black salve” as a “100% cure for skin cancer” [111,112]. The largest issue is there is no correlation between the quality of content and the amount of engagement that content receives [113]. Even if dermatologists developed high-quality educational videos, users may still engage with lower-quality, inaccurate videos, as YouTube offers no verification or credentialing functionality.

Like YouTube, many reviewers found a trend of misinformation, high variability, and low readability on websites. The readability scores of sampled skin cancer websites averaged at the high school level, whereas the recommended readability score for medical information is at the seventh-grade level [114,115].

Misinformation is found across all SM platforms. A review of skin cancer records across Facebook, X, and Pinterest found that 44.7% of records were imprecise and 20% were confusing [116]. The #Stop5G campaign that went viral on X and Facebook broadcasted inaccurate health information, stating that 5G phones were causing skin cancer [117]. Longitudinal melanonychia also went viral on TikTok in 2022. Of the 100 videos examined, only 30% of TikTok postings regarding longitudinal melanonychia encouraged patients to see their physician, and the information was of poor quality as seen by the DISCERN score average of 1.58/5 [118]. Pinterest portrays a low general risk of skin cancer to its users, recommends alternative medicines twice as often as traditional biomedical treatments, and spreads false sunscreen information [119]. Antisunscreen campaigns have become more popular, specifically targeting parents and encouraging homemade sunscreen that is ineffective in protecting the skin [120,121]. Even skin cancer screening examinations, a well-established early detection intervention, are impacted by misinformation: 25% of screening posts on Pinterest were negative, expressing doubts regarding the merit of skin examinations [122]. Facebook support groups may also be poor sources of cancer care information: in one examination of Facebook skin cancer support group comments, 35% of posts had comments that offered medical advice, of which 87% did not align with guideline-concordant care [123].

## Discussion

### Principal Findings

This review has addressed SM’s positive and negative effects on skin cancer. SM drives most persons’ day-to-day communication and can be a powerful tool for health care leaders to communicate important cancer control information. However, communication via SM also introduces the risk of disseminating misinformation. A critical knowledge gap regarding methods to reduce health misinformation within SM has developed. Studies indicate how increasing interactivity and emotions can increase engagement and success of cancer prevention campaigns. Platforms have the potential to disseminate and gather information quickly and to target patients of many demographics. This review identifies the best practices of SM regarding skin cancer and the drawbacks of the ever-changing information environment to help public health figures use SM in the most productive ways and curb the harmful effects of digital media.

### Best Practices

Table 1 is a culmination of the most effective and engaging ways for health officials to use SM to discuss skin cancer. New communication strategies have so much potential and, if used properly, could increase awareness of skin cancer. Many of the studies included in this review attempted to understand the most engaging ways for physicians and researchers to use SM for public health purposes. The most effective strategies use interactivity, emotion, and promotion from a public influencer. Through the education of patients, providers, and other technicians, the opportunity for skin cancer to be caught early and in turn treated easily will increase. Physicians can also use SM to educate themselves on the popular complaints of skin cancer treatments and to understand their patients’ questions and concerns. SM opens a new line of communication that will revolutionize the patient-physician relationship. The affordable nature of the platforms along with the ease of information spread would allow physicians or researchers to easily educate individuals on the best ways to protect themselves from skin cancer and to protect patients from other misinformation across new communication platforms. If public health officials apply these best practices on SM, they can encourage skin health and publicize prevention methods.

**Table 1:** Best practices demonstrating the best ways to increase audience engagement and the educational benefits of social media.

Objective	Best practices
Increase engagement	Interactivity Cognitive dissonance Self-persuasion theory Emotional communication Fear Compassion Humor Shock Influential backing Celebrities Physician credibility (white coat) Legislation blocking indoor tanning
Provide beneficial educational content	Dermatologists to patients Self-skin examinations Prevention information and practices High-risk behaviors Dermatologists to primary care physicians Share the most up-to-date literature Share best practices for prevention education Dermatologist to another technician Hairdressers Nail technicians Tattoo artists Patient to dermatologist Understand the effects of treatments and diseases from the patient’s perspective

### Drawbacks

Limited statistical data regarding user demographics on SM make developing targeted interventions and drawing clear conclusions from SM data mining incomprehensible [126,127]. SM research demographics do not accurately represent the entire patient population with skin cancer. This disables researchers in applying SM trends to the general population with skin cancer, specifically regarding gender or higher education distribution (Table 2) [66].

**Table 2:** A collection of the studies that used SM to recruit participants, broken down by demographics.

	Platform	Responses, n	Female participants, n (%)	Male participants, n (%)	Age (years), mean (SD)	Higher education, n (%)
Strome et al [61]	Unspecified	977	507 (51.9)	470 (48.1)	19.3 (2.4)	—^a^
Al-Atif [5]	WhatsApp	529	466 (88)	63 (12)	36 (10)	449 (87)
Guo et al [59]	WeChat	135	70 (51.9)	65 (48.1)	55.8 (14.2)	—
Telvizian et al [62]	Facebook and X (Twitter)	407	330 (81)	77 (19)	36.2(13.2)	—
David et al [58]	Facebook support groups	214	169 (78.9)	45 (21.1)	40.7 (12.1)	—
Makady et al [60]	Facebook and X (Twitter)	89	62 (69.66)	27 (30.33)	35-64	57 (64)
Wohlk et al [63]	Facebook	561	561 (100)	0 (0)	30	235 (41.8)

^a^Not available.

The educational value of prevention campaigns remains in question. When health care leaders or influencers abuse campaign power, it can reduce the public health campaign’s credibility and effectiveness. While some campaigns have proven effective, there are significant demographic discrepancies in which they reach. These campaigns display a bias toward White individuals, and they cannot significantly reach older individuals or young adults due to ineffective communication methods or minimally engaging content. Campaigns require modification with SM changes to remain relevant and reach all demographics.

The current landscape of skin cancer SM content is poor, and dermatologists’ presence is lacking across platforms. After observing the quality of health care content available to patients, SM cannot be considered a reliable source and should remain unsanctioned by physicians.

Medical misinformation research has demonstrated that the presence of misinformation has increased with new technology. Medical misinformation was extensively studied following the COVID-19 pandemic, and it was found that patients’ trust in misinformation increased as their opinion on public health and medical institutions became more negative [128]. This mistrust may come from the growing influence of misinformation, which may lead patients to resist corrections coming from accredited sources [129]. The challenges seen through this scoping review have mirrored other research findings, showing that web-based platforms pose a challenge due to the ease of distribution of medical misinformation. Furthermore, SM provides a platform for users to share information without consequence or peer review and under the protection of freedom of speech. One pilot study discovered that practitioners encountered misinformation regularly across all specialties. Specifically, they found that 92% of the surveyed dermatologists had encountered medical misinformation presented by their patients [130].

While it is accepted that misinformation is generating obstacles for practitioners, the solution is still heavily debated. To combat misinformation, practitioners must have knowledge of what is being spread to provide their patients with high-quality, evidence-based resources. Through our scoping review of the current SM research environment, we may provide clinicians with an actionable understanding of the current state of SM information. In conjunction, SM platforms and new media technology can adapt content algorithms to modify patterns of misinformation exposure. These platforms could additionally develop technologies that allow users to flag problematic content for other SM users [128].

### Future Research and Interventions

Future research is needed to understand the quality of skin cancer content and develop, implement, and evaluate new prevention campaigns on SM platforms, such as TikTok. The current lack of research on TikTok is alarming, considering the frequency of its use among younger patients. SM requires effective and efficient physician engagement methods to reduce misinformation and promote accurate skin cancer content. Increasing dermatologist engagement could ensure high-quality information and establish credible sources for users. As seen through the studies discussing research recruitment, SM data mining offers enormous opportunities to understand the skin cancer landscape on SM. Future studies using data mining related to skin cancer are needed to understand the scope of skin cancer information across new media.

This review identified specific populations who could benefit from SM interventions, specifically, low SM literate individuals and populations commonly disregarded by prevention campaigns. Increasing SM literacy is one of the most influential methods to ensure users properly digest information and are protected from misinformation. In the past, campaigns and advertisements regarding sun protection have underemphasized people of darker complexion. SM provides an easy, affordable campaign platform to target all audiences. The Dayanara effect [38] and Admassu’s use of Grindr to target sexual minority men [56] demonstrate the credibility of targeting specific audiences through SM. Both campaigns amplified cognizance of skin cancer in communities demographically underrepresented by prevention campaigns. It is essential to diversify our intervention strategies to educate all people who could be diagnosed with skin cancer.

### Limitations

As with all literature reviews, ours is reliant on the quality of the previously published data. Other limitations include word choice and database selection, which inadvertently exclude relevant publications. A language bias may be present, as we excluded all papers for which an English full text could not be identified. Interpretation of data, either our own or that of the original author, potentially risks data misinterpretation. The amount of quantitative data available on this topic was limited, and each study’s variables differed. In addition, much of the research currently involving SM’s effects on skin cancer is contradictory. Some studies conclude that SM has immense potential for prevention, while others argue that it is a source of misinformation. This contradiction was often due to study design or sampling bias by the original authors.

### Conclusions

New communication technology represents both an opportunity to improve public health practices and an obstacle for practitioners to overcome. The full potential of SM has yet to be reached, and health care leaders can make these platforms educational and productive regarding skin cancer prevention. Every day users are at risk for exposure to misinformation, which can decrease their trust in evidence-based medicine and increase their intentions to engage in harmful skin behaviors. This review uncovered the importance of collaboration between health care and SM industries to develop techniques to decrease the spread of misinformation. As SM becomes ubiquitous in society, developing quality strategies that break through and reach target populations becomes essential. Establishing a symbiotic relationship between public health officials and SM communication enables new communication technologies to be used as an accurate source of skin cancer information and could prevent harmful behaviors.

## Appendix

Multimedia Appendix 1 Search strategy.

Multimedia Appendix 2 PRISMA (Preferred Reporting Items for Systematic Reviews and Meta-Analyses) checklist.

Multimedia Appendix 3 STROBE score worksheet used for scoring included papers (scores can be found in Multimedia Appendix 3) [10].

Multimedia Appendix 4 Table of characteristics of all included records.

Multimedia Appendix 5 Ten different studies evaluating YouTube videos for quality (DISCERN, Journal of the American Medical Association, and General Quality Score), understandability (Patient Education Management Assessment Tool-Understandability), and actionability (Patient Education Management Assessment Tool-Actionability) of videos on skin cancer topics.

## Abbreviations

PRISMA: Preferred Reporting Items for Systematic Reviews and Meta-Analyses
SM: social media
STROBE: Strengthening the Reporting of Observational Studies in Epidemiology

## References

[1] (2023). Global digital population as of April 2023. Statista.

[2] Jha A, Lin L, Savoia E (2016). The use of social media by state health departments in the US: analyzing health communication through Facebook. J Community Health.

[3] Braun LA, Zomorodbakhsch B, Keinki C, Huebner J (2019). Information needs, communication and usage of social media by cancer patients and their relatives. J Cancer Res Clin Oncol.

[4] Mheidly N, Fares J (2020). Leveraging media and health communication strategies to overcome the COVID-19 infodemic. J Public Health Policy.

[5] Al-Atif HM (2021). A cross-sectional survey of knowledge of skin cancer in Saudi Arabia. Dermatol Pract Concept.

[6] Rahib L, Wehner MR, Matrisian LM, Nead KT (2021). Estimated projection of US cancer incidence and death to 2040. JAMA Netw Open.

[7] Arksey H, O'Malley L (2005). Scoping studies: towards a methodological framework. Int J Soc Res Methodol.

[8] Levac D, Colquhoun H, O'Brien KK (2010). Scoping studies: advancing the methodology. Implement Sci.

[9] Moher D, Liberati A, Tetzlaff J, Altman DG, PRISMA Group (2009). Preferred reporting items for systematic reviews and meta-analyses: the PRISMA statement. PLoS Med.

[10] Cuschieri S (2019). The STROBE guidelines. Saudi J Anaesth.

[11] Kim HM, Saffer AJ, Liu W, Sun J, Li Y, Zhen L, Yang A (2022). How public health agencies break through COVID-19 conversations: a strategic network approach to public engagement. Health Commun.

[12] Southwell BG, Brennen JSB, Paquin R, Boudewyns V, Zeng J (2022). Defining and measuring scientific misinformation. Ann Am Acad Pol Soc Sci.

[13] Jhawar N, Lipoff JB (2019). Variable potential for social media platforms in raising skin cancer awareness. Dermatology.

[14] Wei C, Allais B, Tornberg HN, Quan T, Adusumilli NC, Patel VA, Friedman AJ (2021). The utilization of the Altmetric and PlumX scores in evaluating the top 100 trending melanoma articles in social media. J Am Acad Dermatol.

[15] Jain N, Zachary I, Boren S (2022). Who influences cancer conversations on Twitter?: A comparative surveillance of cancer communications. Stud Health Technol Inform.

[16] Gough A, Hunter RF, Ajao O, Jurek A, McKeown G, Hong J, Barrett E, Ferguson M, McElwee G, McCarthy M, Kee F (2017). Tweet for behavior change: using social media for the dissemination of public health messages. JMIR Public Health Surveill.

[17] Murthy D, Eldredge M (2016). Who tweets about cancer? An analysis of cancer-related tweets in the USA. Digit Health.

[18] Gomaa BT, Walsh-Buhi ER, Funk RJ (2022). Understanding melanoma talk on Twitter: the lessons learned and missed opportunities. Int J Environ Res Public Health.

[19] Kassamali B, Villa-Ruiz C, Mazori DR, Min M, Cobos GA, LaChance AH (2021). Characterizing top educational TikTok videos by dermatologists in response to "TikTok and dermatology: an opportunity for public health engagement". J Am Acad Dermatol.

[20] Villa-Ruiz C, Kassamali B, Mazori DR, Min M, Cobos G, LaChance A (2021). Overview of TikTok's most viewed dermatologic content and assessment of its reliability. J Am Acad Dermatol.

[21] Harp T, Rundle CW, Anderson J, Presley C, Concilla A, Laughter M, Dellavalle RP (2022). An analysis of sunscreen-related hashtags on Instagram. Photodermatol Photoimmunol Photomed.

[22] Gomaa B, Houghton RF, Crocker N, Walsh-Buhi ER (2022). Skin cancer narratives on Instagram: content analysis. JMIR Infodemiology.

[23] Cho H, Silver N, Na K, Adams D, Luong KT, Song C (2018). Visual cancer communication on social media: an examination of content and effects of #Melanomasucks. J Med Internet Res.

[24] Nosrati A, Pimentel MA, Falzone A, Hegde R, Goel S, Chren MM, Eye R, Linos E, Pagoto S, Walkosz BJ (2018). Skin cancer prevention messages on Facebook: likes, shares, and comments. J Am Acad Dermatol.

[25] Morrison L, Chen C, Torres JS, Wehner M, Junn A, Linos E (2019). Facebook advertising for cancer prevention: a pilot study. Br J Dermatol.

[26] Noar SM, Leas E, Althouse BM, Dredze M, Kelley D, Ayers JW (2018). Can a selfie promote public engagement with skin cancer?. Prev Med.

[27] Coups EJ, Manne SL, Pagoto SL, Criswell KR, Goydos JS (2018). Facebook intervention for young-onset melanoma patients and their family members: pilot and feasibility study. JMIR Dermatol.

[28] Niu Z, Willoughby JF, Coups EJ, Stapleton JL (2021). Effects of website interactivity on skin cancer-related intentions and user experience: factorial randomized experiment. J Med Internet Res.

[29] Olayiwola O, Lazovich D, Wipf A, Goldfarb N, Lindgren B, Bellefeuille G, Farah RS (2021). The use of the video, "Dear 16-Year-Old Me," as a melanoma education tool in ambulatory dermatology. Dermatol Surg.

[30] Myrick JG, Oliver MB (2015). Laughing and crying: mixed emotions, compassion, and the effectiveness of a YouTube PSA about skin cancer. Health Commun.

[31] Potente S, McIver J, Anderson C, Coppa K (2011). "It's a Beautiful Day ... for Cancer": an innovative communication strategy to engage youth in skin cancer prevention. Soc Mark Q.

[32] Hughes-Barton D, Hutchinson A, Prichard I, Wilson C (2021). Acceptability of online sun exposure awareness-raising interventions among young Australian women: an exploratory mixed-methods study. Health Promot Int.

[33] Peconi J, Wright S, Carter A, Da Roza C, Eden-Davies C, Frame R, Mughal AA (2019). Don't be a lobster: a novel way of promoting sun protection on Welsh beaches. Br J Dermatol.

[34] Nguyen J, Gilbert L, Priede L, Heckman C (2019). The reach of the "Don't Fry Day" Twitter campaign: content analysis. JMIR Dermatol.

[35] Nguyen JL, Heckman C, Perna F (2018). Analysis of the Twitter "Don't Fry Day" campaign. JAMA Dermatol.

[36] Rahmani G, McArdle A, Kelly JL (2018). The Hugh Jackman effect—the impact of celebrity health disclosure on skin cancer awareness. Dermatol Surg.

[37] Pavelko RL, Myrick JG, Verghese RS, Hester JB (2017). Public reactions to celebrity cancer disclosures via social media: implications for campaign message design and strategy. Health Educ J.

[38] Srivastava R, Wassef C, Rao BK (2019). The Dayanara effect: increasing skin cancer awareness in the Hispanic community. Cutis.

[39] Correia O, Duarte AF, Del Marmol V, Picoto A (2018). Euromelanoma in Portugal. How useful was the Euromelanoma campaign between 2010 and 2017?. Int J Dermatol.

[40] Myrick JG, Waldron KA, Cohen O, DiRusso C, Shao R, Cho E, Willoughby JF, Turrisi R (2022). The effects of embedded skin cancer interventions on sun-safety attitudes and attention paid to tan women on Instagram. Front Psychol.

[41] Mingoia J, Hutchinson AD, Gleaves DH, Wilson C (2019). The impact of a social media literacy intervention on positive attitudes to tanning: a pilot study. Comput Hum Behav.

[42] Mingoia J, Hutchinson AD, Gleaves DH, Wilson C (2020). Does better media literacy protect against the desire for tanned skin and propensity for making appearance comparisons?. Soc Media Soc.

[43] Dawson AL, Hay AA, Huff LS, Gamble RG, Howe W, Kane I, Dellavalle RP (2011). Online videos to promote sun safety: results of a contest. Dermatol Rep.

[44] Nabi RL, Huskey R, Nicholls SB, Keblusek L, Reed M (2019). When audiences become advocates: self-induced behavior change through health message posting in social media. Comput Hum Behav.

[45] Pagoto SL, Waring ME, Groshon LC, Rosen AO, Schroeder MW, Goetz JM (2022). Proof-of-concept feasibility trial of a dissonance-based sun safety intervention for young adult tanners. Ann Behav Med.

[46] Køster B, Thorgaard C, Philip A, Clemmensen H (2011). Sunbed use and campaign initiatives in the Danish population, 2007-2009: a cross-sectional study. J Eur Acad Dermatol Venereol.

[47] Buller DB, Pagoto S, Baker K, Walkosz BJ, Hillhouse J, Henry KL, Berteletti J, Bibeau J (2021). Results of a social media campaign to prevent indoor tanning by teens: a randomized controlled trial. Prev Med Rep.

[48] Griffin L, Roche D, Roche L, Murphy M (2018). Local radio and local newspaper best methods to reach older male population for Euromelanoma campaign in Ireland. J Eur Acad Dermatol Venereol.

[49] Marchetti MA, Sar-Graycar L, Dusza SW, Nanda JK, Kurtansky N, Rotemberg VM, Hay JL (2022). Prevalence and age-related patterns in health information-seeking behaviors and technology use among skin cancer survivors: survey study. JMIR Dermatol.

[50] O'Bryan C, Gao DX, Kentosh JB (2022). Impact of free skin screenings on number of biopsy-confirmed skin cancers and analysis of popular advertising methods: a 4-year retrospective study at a single dermatology practice. Int J Dermatol.

[51] Agha-Mir-Salim L, Bhattacharyya A, Hart D, Lewandowska M, Spyropoulou E, Stinson L, Tiefenbach J (2020). A randomised controlled trial evaluating the effectiveness of Facebook compared to leaflets in raising awareness of melanoma and harmful sun-related behaviour among young adults. Eur J Cancer Prev.

[52] McLoone JK, Meiser B, Karatas J, Chau J, Zilliacus E, Kasparian N (2012). Perceptions of melanoma risk among Australian adolescents: barriers to sun protection and recommendations for improvement. Asia-Pac J Clin Oncol.

[53] Park SE, Tang L, Bie B, Zhi D (2019). All pins are not created equal: communicating skin cancer visually on Pinterest. Transl Behav Med.

[54] Grewal SK, Reddy V, Tomz A, Lester J, Linos E, Lee PK (2021). Skin cancer in skin of color: a cross-sectional study investigating gaps in prevention campaigns on social media. J Am Acad Dermatol.

[55] de Vere Hunt I, Owen S, Amuzie A, Nava V, Tomz A, Barnes L, Robinson JK, Lester J, Swetter S, Linos E (2023). Qualitative exploration of melanoma awareness in black people in the USA. BMJ Open.

[56] Admassu N, Pimentel MA, Halley MC, Torres J, Pascua N, Katz KA, Linos E (2019). Motivations among sexual-minority men for starting and stopping indoor tanning. Br J Dermatol.

[57] Admassu NE (2018). Sexual minority men and indoor tanning: a qualitative analysis of social media engagement and perceptions of public health advertising [thesis]. Univeristy of California.

[58] David MP, Funderburg A, Selig JP, Brown R, Caliskan PM, Cove L, Dicker G, Hoffman L, Horne T, Gardner JM (2019). Perspectives of patients with dermatofibrosarcoma protuberans on diagnostic delays, surgical outcomes, and nonprotuberance. JAMA Netw Open.

[59] Guo Y, Shen M, Zhang X, Xiao Y, Zhao S, Yin M, Bu W, Wang Y, Chen X, Su J (2021). Unemployment and health-related quality of life in melanoma patients during the COVID-19 pandemic. Front Public Health.

[60] Makady A, Kalf RRJ, Ryll B, Spurrier G, de Boer A, Hillege H, Klungel OH, Goettsch W (2018). Social media as a tool for assessing patient perspectives on quality of life in metastatic melanoma: a feasibility study. Health Qual Life Outcomes.

[61] Strome A, Chang T, Waselewski M, Lamberg O, Herbert K (2022). Skin cancer prevention: knowledge and perceptions of a nationwide sample of youth. Ann Fam Med.

[62] Telvizian T, Al Ghadban Y, Alawa J, Mukherji D, Zgheib NK, Sawaf B, Nasr R, Bardus M (2021). Knowledge, beliefs, and practices related to cancer screening and prevention in Lebanon: community and social media users' perspectives. Eur J Cancer Prev.

[63] Wøhlk IMR, Philipsen PA, Wulf HC (2016). Factors associated with cessation of sunbed use among Danish women. Photodermatol Photoimmunol Photomed.

[64] Faust G, Booth A, Merinopoulou E, Halhol S, Tosar H, Nawaz A, Szlachetka M, Chiu G (2022). The experiences of patients with adjuvant and metastatic melanoma using disease-specific social media communities in the advent of novel therapies (excite project): social media listening study. JMIR Cancer.

[65] McDonald L, Behl V, Sundar V, Mehmud F, Malcolm B, Ramagopalan S (2019). Validity of social media for assessing treatment patterns in oncology patients: a case study in melanoma. JAMIA Open.

[66] Radzikowski JR, Hollen H, Fuhrmann S (2015). Using Twitter content to crowdsource opinions on tanning in the United States. J Cartogr Geogr inf.

[67] Waring ME, Baker K, Peluso A, May CN, Pagoto SL (2019). Content analysis of Twitter chatter about indoor tanning. Transl Behav Med.

[68] Tivey A, Huddar P, Shotton R, Cheese I, Daniels S, Lorigan P, Lee RJ (2021). Patient engagement in melanoma research: from bench to bedside. Future Oncol.

[69] Dellavalle R, Endly D, Sampson B (2012). Journal of the American Academy of Dermatology inaugural year Facebook posting metrics. J Invest Dermatol.

[70] Karimkhani C, Gamble R, Dellavalle R (2014). Social media impact factor: the top ten dermatology journals on facebook and twitter. Dermatology Online J.

[71] Karimkhani C, Connett J, Boyers L, Quest T, Dellavalle RP (2014). Dermatology on Instagram. Dermatology Online J.

[72] Hay AA, Gamble RG, Huff LS, Dellavalle RP (2011). Internet social networking sites and the future of dermatology journals: promises and perils. J Am Acad Dermatol.

[73] Amir M, Sampson BP, Endly D, Tamai JM, Henley J, Brewer AC, Dunn JH, Dunnick CA, Dellavalle RP (2014). Social networking sites: emerging and essential tools for communication in dermatology. JAMA Dermatol.

[74] Ashack KA, Burton KA, Dellavalle RP (2016). Dermatology in Doximity. Dermatol Online J.

[75] Pemmaraju N, Thompson MA, Mesa RA, Desai T (2017). Analysis of the use and impact of Twitter during American Society of Clinical Oncology annual meetings from 2011 to 2016: focus on advanced metrics and user trends. J Oncol Pract.

[76] Joly-Chevrier M, Aly S, Lefrançois P (2022). Comparison of basal cell carcinoma posts, comments and authors between Reddit and Quora forums. J Cutan Med Surg.

[77] Damude S, Hoekstra-Weebers JEHM, van Leeuwen BL, Hoekstra HJ (2017). Melanoma patients' disease-specific knowledge, information preference, and appreciation of educational YouTube videos for self-inspection. Eur J Surg Oncol.

[78] Machado CK, Haddad A, Santos IDAO, Ferreira LM (2021). "Pele alerta project": prevention and early detection of skin cancer aimed at beauty professionals. Rev Bras Cir Plast.

[79] Gonzalez CD, Pona A, Walkosz BJ, Dellavalle RP (2019). Hispanic tattoo artists could provide skin cancer prevention via aftercare instructions and social media. J Drugs Dermatol.

[80] Kamath P, Kursewicz C, Ingrasci G, Jacobs R, Agarwal N, Nouri K (2019). Analysis of patient perceptions of Mohs surgery on social media platforms. Arch Dermatol Res.

[81] Kalf RRJ, Delnoij DMJ, Ryll B, Bouvy ML, Goettsch WG (2021). Information patients with melanoma spontaneously report about health-related quality of life on web-based forums: case study. J Med Internet Res.

[82] Chauhan J, Aasaithambi S, Márquez-Rodas I, Formisano L, Papa S, Meyer N, Forschner A, Faust G, Lau M, Sagkriotis A (2022). Understanding the lived experiences of patients with melanoma: real-world evidence generated through a European social media listening analysis. JMIR Cancer.

[83] Maganty N, Ilyas M, Ginsberg Z, Sharma A (2018). Social media as a platform for information and support for melanoma patients: analysis of melanoma Facebook groups and pages. JMIR Dermatol.

[84] Vraga EK, Bode L, Tully M (2022). The effects of a news literacy video and real-time corrections to video misinformation related to sunscreen and skin cancer. Health Commun.

[85] Mingoia J, Hutchinson AD, Gleaves DH, Corsini N, Wilson C (2017). Use of social networking sites and associations with skin tone dissatisfaction, sun exposure, and sun protection in a sample of Australian adolescents. Psychol Health.

[86] Myrick JG, Noar SM, Kelley D, Zeitany AE (2017). The relationships between female adolescents' media use, indoor tanning outcome expectations, and behavioral intentions. Health Educ Behav.

[87] Stapleton JL, Hillhouse J, Coups EJ, Pagoto S (2016). Social media use and indoor tanning among a national sample of young adult nonHispanic white women: a cross-sectional study. J Am Acad Dermatol.

[88] Moreno MA, Jenkins MC, Lazovich D (2021). Tanning misinformation posted by businesses on social media and related perceptions of adolescent and young adult White non-Hispanic women: mixed methods study. JMIR Dermatol.

[89] Ricklefs CA, Asdigian NL, Kalra HL, Mayer JA, Dellavalle RP, Holman DM, Crane LA (2016). Indoor tanning promotions on social media in six US cities #UVTanning #tanning. Transl Behav Med.

[90] Jenkins M, Lazovich D, Moreno MA (2019). #Paleshaming: social media messages and adolescents' perceptions. J Adolesc Health.

[91] Fogel J, Krausz F (2013). Watching reality television beauty shows is associated with tanning lamp use and outdoor tanning among college students. J Am Acad Dermatol.

[92] Banerjee SC, Rodríguez VM, Greene K, Hay JL (2019). Trending on Pinterest: an examination of pins about skin tanning. Transl Behav Med.

[93] Hossler EW, Conroy MP (2008). YouTube as a source of information on tanning bed use. Arch Dermatol.

[94] Kream EJ, Watchmaker JD, Dover JS (2022). TikTok sheds light on tanning: tanning is still popular and emerging trends pose new risks. Dermatol Surg.

[95] Stekelenburg N, Horsham C, O'Hara M, Janda M (2020). Using social media to determine the affective and cognitive components of tweets about sunburn. Dermatology.

[96] Wehner MR, Chren MM, Shive ML, Resneck JS, Pagoto S, Seidenberg AB, Linos E (2014). Twitter: an opportunity for public health campaigns. Lancet.

[97] DeBiasio C, Li HOY, Brandts-Longtin O, Kirchhof MG (2022). Cannabis use in dermatology: a cross-sectional study of YouTube videos. J Cutan Med Surg.

[98] Ruppert L, Koster B, Siegert AM, Cop C, Boyers L, Karimkhani C, Winston H, Mounessa J, Dellavalle RP, Reinau D, Diepgen T, Surber C (2017). YouTube as a source of health information: analysis of sun protection and skin cancer prevention related issues. Dermatology.

[99] Guzman AK, Wang RH, Nazarian RS, Barbieri JS (2020). Evaluation of YouTube as an educational resource for treatment options of common dermatologic conditions. Int J Dermatol.

[100] Huang CM, Li HOY, Macdonald J (2021). YouTube as a source of patient information for Mohs micrographic surgery: a systematic analysis. Dermatol Surg.

[101] Iglesias-Puzas Á, Conde-Taboada A, López-Bran E (2022). A cross-sectional study of YouTube videos on Mohs surgery: quality of content and sentiment analysis. J Am Acad Dermatol.

[102] Joly-Chevrier M, Aly S, Lefrançois P (2023). Quality assessment of skin cancer videos calls for improved patient content: a YouTube cross-sectional study. J Cutan Med Surg.

[103] Mamo A, Szeto MD, Mirhossaini R, Fortugno A, Dellavalle RP (2021). Tetrahydrocannabinol and skin cancer: analysis of YouTube videos. JMIR Dermatol.

[104] Reinhardt L, Steeb T, Harlaß M, Brütting J, Meier F, Berking C (2022). Are YouTube videos on cutaneous squamous cell carcinoma a useful and reliable source for patients?. J Dtsch Dermatol Ges.

[105] Reinhardt L, Steeb T, Mifka A, Berking C, Meier F, German Skin Cancer Council (2023). Quality, understandability and reliability of YouTube videos on skin cancer screening. J Cancer Educ.

[106] Steeb T, Reinhardt L, Görgmayr C, Weingarten H, Doppler A, Brütting J, Meier F, Berking C (2020). German YouTube™ videos as a source of information on cutaneous melanoma: a critical appraisal. J Eur Acad Dermatol Venereol.

[107] Steeb T, Reinhardt L, Harlaß M, Heppt MV, Meier F, Berking C (2022). Assessment of the quality, understandability, and reliability of YouTube videos as a source of information on basal cell carcinoma: web-based analysis. JMIR Cancer.

[108] Boyers LN, Quest T, Karimkhani C, Connett J, Dellavalle RP (2014). Dermatology on YouTube. Dermatol Online J.

[109] St Claire KM, Rietcheck HR, Patel RR, Dunnick C, Dellavalle RP (2018). Dermatology on YouTube—an update and analysis of new trends. Dermatol Online J.

[110] Quinn EK, Fenton S, Ford-Sahibzada CA, Harper A, Marcon AR, Caulfield T, Fazel SS, Peters CE (2022). COVID-19 and vitamin D misinformation on YouTube: content analysis. JMIR Infodemiology.

[111] Basch CH, Basch CE, Hillyer GC, Reeves R (2015). YouTube videos related to skin cancer: a missed opportunity for cancer prevention and control. JMIR Cancer.

[112] Babamiri K, Nassab RS (2010). The availability and content analysis of melanoma information on YouTube. Plast Reconstr Surg.

[113] Smeeton B, Wormald JCR, Plonczak AM, Butler D, Hamilton S (2018). A critical review of melanoma self-screening tools on YouTube—a missed opportunity?. J Plast Reconstr Aesthet Surg.

[114] Alshaikh EA, Almedimigh AF, Alruwaili AM, Almajnoni AH, Alhajiahmed A, Almalki TS, Alfaraj SZ, Pines JM (2019). Patient-focused online resources for melanoma: highly variable content and quality. J Cancer Educ.

[115] Özistanbullu D, Weber R, Kleemann J, Jäger M, Kippenberger S, Kaufmann R, Meissner M (2022). Exploring the most visible websites on cutaneous T-cell lymphoma-revealing limited quality of patient health information on the internet. J Eur Acad Dermatol Venereol.

[116] Iglesias-Puzas Á, Conde-Taboada A, Aranegui-Arteaga B, López-Bran E (2021). "Fake news" in dermatology. Results from an observational, cross-sectional study. Int J Dermatol.

[117] Rafferty S, O'Connor C, Murphy M (2021). "Fake news"-5G mobile phones and skin cancer: a global analysis of concerns on social media. Skin Res Technol.

[118] Albucker SJ, Lipner SR (2023). Social media creators are far from nailing it: a cross-sectional analysis of 100 longitudinal melanonychia TikTok videos shows poor educational content and lack of skin of color representation. J Cutan Med Surg.

[119] Tang L, Park SE (2017). Sun exposure, tanning beds, and herbs that cure: an examination of skin cancer on Pinterest. Health Commun.

[120] Merten JW, Roberts KJ, King JL, McKenzie LB (2020). Pinterest homemade sunscreens: a recipe for sunburn. Health Commun.

[121] Tamminga MA, Lipoff JB (2021). Understanding sunscreen and photoprotection misinformation on parenting blogs: a mixed-method study. Pediatr Dermatol.

[122] Merten J, King J, Dedrick A (2022). Content analysis of skin cancer screenings on Pinterest: an exploratory study. Int J Environ Res Public Health.

[123] Petukhova TA, Wilson BN, Gadjiko M, Lee EH, Wang J, Rossi AM, Nehal KS (2020). Utilization of Facebook for support and education by patients with skin cancer. Dermatology Online J.

[124] (2023). How many people use TikTok?. Oberlo.

[125] (2020). The Melanoma Research Foundation and Dayanara Torres want you to #GetNaked!. Melanoma Research Foundation.

[126] Blee I, Kumar S, Dhariwal S, Tso S (2020). Methodological considerations when exploring the impact of social media health promotional materials. Clin Exp Dermatol.

[127] Guckian J, Jobling K, Oliphant T, Weatherhead S, Blasdale K (2020). 'I saw it on Facebook!' Assessing the influence of social media on patient presentation to a melanoma screening clinic. Clin Exp Dermatol.

[128] Southwell BG, Niederdeppe J, Cappella JN, Gaysynsky A, Kelley DE, Oh A, Peterson EB, Chou WYS (2019). Misinformation as a misunderstood challenge to public health. Am J Prev Med.

[129] Thorson EA, Sheble L, Southwell BG, Southwell BG, Thorson EA, Sheble L (2018). An agenda for misinformation research. Misinformation and Mass Audiences.

[130] Wood JL, Lee GY, Stinnett SS, Southwell BG (2021). A pilot study of medical misinformation perceptions and training among practitioners in North Carolina (USA). Inquiry.

