# Supplementary material for: The New Media Landscape and Its Effects on Skin Cancer Diagnostics, Prognostics, and Prevention: Scoping Review
Source: JMIR Dermatol. 2024 Apr 8;7:e53373. doi: 10.2196/53373 (PMC11036192; doi:10.2196/53373)
Supplement: Multimedia Appendix 1 [file derma_v7i1e53373_app1.pdf]

THE UNIVERSITY OF TEXAS
MD Anderson Cancer Center
Making Cancer History®

Research Medical Library
Access. Answers. Expertise.

David P. Farris, MSIS, AHIP
Librarian, Research Services
dpfarris@mdanderson.org
T 713-745-8929

#	Searches	Results
1	exp Skin Neoplasms/	143018
2	exp Melanoma/	108557
3	(acanthoma* or "basal cell carcinoma*" or "dermatolog* problem*" or dermoscop* or melanoma* or "Muir-Torre Syndrome" or "pigmented lesion*" or "skin disorder*" or "skin lesion*" or "squamous cell carcinoma*").ti,ab.	307153
4	((cutaneous or skin or "sebaceous gland" or "sweat gland") adj2 (cancer* or carcinoma* or neoplasm*)).ti,ab.	33598
5	or/1-4 [Melanoma]	407986
6	Social Media/	15686
7	(Twitter or tweet* or Facebook or "Face book" or TikTok or "Tik-Tok" or Flickr or Instagram or LinkedIn or MySpace or Pinterest or Reddit or Snapchat or WeChat or "We-Chat" or WhatsApp or YouTube or "You Tube" or Tumblr or hashtag or "hash tag" or "social media*" or "web 2.0" or "web2.0").mp.	43813
8	or/6-7 [Social Media]	43813
9	5 and 8 [Melanoma + Social Media]	168
10	("28818251" or "29500110" or "29732480" or "30185403" or "36141558" or "36862030").ui.	6
11	9 or 10 [Results + Key Articles]	168
12	11 not 9 [Missing Key Articles]	0
