# Supplementary material for: The New Media Landscape and Its Effects on Skin Cancer Diagnostics, Prognostics, and Prevention: Scoping Review
Source: JMIR Dermatol. 2024 Apr 8;7:e53373. doi: 10.2196/53373 (PMC11036192; doi:10.2196/53373)
Supplement: Multimedia Appendix 4 [file derma_v7i1e53373_app4.pdf]

Author/ year	Study type	Geographic location	Platform	Category	Strobe score
Al-Atif (2021) [5]	Cross-sectional survey	Saudi Arbia	unspecified	Research	18
Jhawar and Lipoff (2019) [13]	Cross-sectional analysis	United States	Faceboook, Twitter, Instagram	Engagement	16
Wei et al (2021) [14]	Content Analysis		Twitter	Engagement	17
Jain et al (2022) [15]	Content Analysis	United States	Twitter	Engagement	18
Gough et al (2017) [16]	Feasibility study	United Kingdom	Twitter	Engagement	20
Murthy and Eldredge (2016) [17]	Content Analysis	United States	Twitter	Engagement	19
Gomaa et al (2022) [18]	Content Analysis		Twitter	Engagement	20
Kassamali et al (2021) [19]	Cross-sectional analysis		TikTok	Engagement	16
Villa-Ruiz et al (2021) [20]	Cross-sectional analysis		TikTok	Engagement	17
Harp et al (2022) [21]	Cross-sectional analysis		Instagram	Engagement	19
Gomaa et al (2022) [22]	Content Analysis		Instagram	Engagement	20
Cho et al (2018) [23]	Content Analysis		Instagram	Engagement	20
Nosrati et al (2018) [24]	Cross-sectional analysis		Facebook	Engagement	17
Morrison et al (2019) [25]	Feasibility study	united states	Facebook	Engagement	15
Noar et al (2018) [26]	Observational analysis		Facebook and google	Engagement	18
Coups et al (2018) [27]	Feasibility study	United States	Facebook	Engagement	21
Niu et al (2021) [28]	Factorial randomized experiment		Websites	Engagement	20
Olayiwola et al (2021) [29]	Interventional study	United States	YouTube	Campaigns	19
Myrick and Oliver (2015) [30]	Interventional study		YouTube	Campaigns	20
Potente et al (2011) [31]	Observational study	Australia	YouTube	Campaigns	16

Hughes-Barton et al (2021) [32]	Exploratory mixed methods study	Australia	YouTube	Campaigns	20
Peconi et al (2018) [33]	Interventional study	United Kingdom	YouTube	Campaigns	14
Nguyen et al (2019) [34]	Interventional study		Facebook and YouTube	Campaigns	19
Nguyen et al (2018) [35]	Content Analysis		Twitter	Campaigns	13
Rahmani et al (2018) [36]	Observational study		Google	Campaigns	13
Pavelko et al (2017) [37]	Content Analysis		Facebook	Campaigns	18
Srivastava et al (2019) [38]	Cohort Study		Instagram	Campaigns	13
Correia et al (2018) [39]	Experimental study	Portugal	unspecified	Campaigns	15
Myrick et al (2022) [40]	Interventional study	United States	Instagram	Campaigns	20
Mingoia et al (2019) [41]	Feasibility study	Australia	Facebook	Campaigns	20
Mingoia et al (2020) [42]	interventional study	Australia	Facebook and Instagram	Campaigns	20
Dawson et al (2011) [43]	Content Analysis		YouTube	Campaigns	18
Nabi et al (2019) [44]	Survey study	United States	Facebook, Twitter, and blogs	Campaigns	19
Pagoto et al (2022) [45]	Feasibility study		Facebook	Campaigns	21
Koster et al (2011) [46]	Cross-sectional analysis	Denmark	Facebook, Myspace, and YouTube	Campaigns	21
Buller et al (2021) [47]	Randomized control trial	United States	Facebook	Campaigns	22
Griffin et al (2018) [48]	Explanatory survey	Ireland	Facebook and Twitter	Demographics	15
Marchetti et al (2022) [49]	Survey study		unspecified	Demographics	20
O'Bryan et al (2022) [50]	Retrospective study	United States	unspecified	Demographics	14
Agha-Mir-Salim et al (2020) [51]	Randomized control trial	United Kingdom	Facebook	Demographics	19
McLoone et al (2012) [52]	Qualitative analysis	Australia	Facebook and Twitter	Demographics	19

Park et al (2019) [53]	Descriptive Content Analysis		Pinterest	Demographics	20
Grewal et al (2021) [54]	Cross-sectional analysis		Facebook, Instagram, and Twitter	Demographics	18
De Vere Hunt et al (2023) [55]	Qualitative analysis	United States	Facebook	Demographics	21
Admassu et al (2019) [56]	Qualitative analysis		unspecified	Demographics	17
Admassu (2018) [57]	Qualitative analysis	United States	Facebook	Demographics	20
David et al (2019) [58]	Survey study	United States, United Kingdom, Canada, Australia, India, Italy, Germany	Facebook	Research	20
Guo et al (2021) [59]	Cross-sectional analysis	China	unspecified	Research	20
Makady et al (2018) [60]	Feasbility study	United Kingdom	Facebook, Twitter, linkedin	Research	21
Strome et al (2022) [61]	Exploratory Survey		unspecified	Research	12
Telvizian et al (2021) [62]	Cross-sectional analysis	Lebanon	Facebook and Twitter	Research	19
Wohlk et al (2016) [63]	Survey Study	Netherlands	Facebook	Research	18
Faust et al (2022) [64]	Retrospective review		unspecified	Research	22
McDonald et al (2019) [65]	Retrosepective study		Facebook, Twitter, YouTube, blogs	Research	20
Radzikowski et al (2015) [66]	Content Analysis		Twitter	Research	19
Waring et al (2019) [67]	Cross-sectional Content Analysis		Twitter	Research	19
Tivey et al (2021) [68]	Systematic Review		unspecified	Research	19
Dellavalle et al (2012) [69]	Content Analysis		Facebook	Education	16
Karimkhani et al (2014) [70]	Content Analysis		Facebook	Education	17
Karimkhani et al (2014) [71]	Content Analysis		Instagram	Education	17

Hay et al (2011) [72]	Content Analysis		YouTube, Twitter, and Facebook	Education	17
Amir et al (2014) [73]	Cross-sectional analysis		Facebook, Twitter, linkedin	Education	19
Ashack et al (2016) [74]	Retrospective review	United States	Doximity	Education	18
Pemmaraju et al (2017) [75]	Retrospective review		Twitter	Education	20
Joly-Chevrier et al (2022) [76]	Retrospective review		Reddit and Quora	Education	15
Damude et al (2017) [77]	Descriptive survey	Netherlands	YouTube	Education	18
Machado et al (2021) [78]	Article	Brazil	YouTube	Education	16
Gonzalez et al (2019) [79]	Semi-structured interview study	United States	Facebook, Instagram, Twitter, snapchat, Pinterest, YouTube	Education	17
Kamath et al (2019) [80]	Content Analysis		Instagram and Twitter	Patient Support	19
Kalf et al (2021) [81]	Survey study	United States and United Kingdom	Google and Bing (blogs)	Patient Support	20
Chauhan et al (2022) [82]	Retrospective review	Europe	Twitter	Patient Support	21
Maganty et al (2018) [83]	Systematic analysis		Facebook	Patient Support	19
Vraga et al (2022) [84]	Interventional experiment		Facebook	Misinformation	20
Mingoia et al (2017) [85]	exploratory survey	Australia	Facebook, Twitter, Instagram, Snapchat, tumblr, flickr, and myspace	Misinformation	20
Myrick et al (2017) [86]	Survey study	United States	unspecified	Misinformation	20
Stapleton et al (2016) [87]	Cross-sectional analysis		Facebook, Twitter, Instagram, and Pinterest	Misinformation	15

Moreno et al (2021) [88]	Mixed-methods study		Facebook	Misinformation	20
Ricklefs et al (2016) [89]	Observational study		Twitter and Facebook	Misinformation	20
Jenkins et al (2019) [90]	Mixed-methods analysis		unspecified	Misinformation	16
Fogel and Krausz (2013) [91]	Survey study	United States	unspecified	Misinformation	18
Banerjee et al (2019) [92]	Quantitative content analysis	United States	Pinterest	Misinformation	21
Hossler and Conroy (2008) [93]	Cross-sectional analysis		YouTube	Misinformation	16
Kream et al (2022) [94]	Cross-sectional analysis		TikTok	Misinformation	18
Stekelenburg et al (2020) [95]	Qualitative Content Analysis	Switzerland	Twitter	Misinformation	18
Wehner et al (2014) [96]	Content Analysis		Twitter	Misinformation	13
DeBiasio et al (2022) [97]	Cross-sectional analysis		YouTube	Misinformation	15
Ruppert et al (2017) [98]	Content Analysis		YouTube	Misinformation	18
Guzman et al (2020) [99]	Content Analysis		YouTube	Misinformation	17
Huang et al (2021) [100]	Systematic cross-sectional analysis		YouTube	Misinformation	19
Iglesias-Puzas et al (2021) [101]	Sentiment analysis		YouTube	Misinformation	18
Joly-Chevrier et al (2023) [102]	Cross-sectional analysis		YouTube	Misinformation	16
Mamo et al (2021) [103]	Content Analysis		YouTube	Misinformation	19
Reinhardt et al (2022) [104]	Retrospective review		YouTube	Misinformation	17
Reinhardt et al (2023) [105]	Retrospective review		YouTube	Misinformation	20
Steeb et al (2020) [106]	Content Analysis	Germany	YouTube	Misinformation	16
Steeb et al (2022) [107]	Content Analysis		YouTube	Misinformation	21
Boyers et al (2014) [108]	Content Analysis		YouTube	Misinformation	15

St Claire et al (2018) [109]	Content Analysis		YouTube	Misinformation	17
Quinn et al (2022) [110]	Qualitative Content Analysis		YouTube	Misinformation	20
Basch et al (2015) [111]	Content Analysis		YouTube	Misinformation	20
Babamiri and Nassab (2010) [112]	Content Analysis		YouTube	Misinformation	16
Smeeton et al (2018) [113]	Retrospective review		YouTube	Misinformation	14
Alshaikh et al (2019) [114]	Descriptive study	United States	Internet	Misinformation	19
Ozistanbullu et al (2022) [115]	Content Analysis		unspecified	Misinformation	18
Iglesias-Puzas et al (2021) [116]	Observational, cross-sectional study		Facebook, Pinterest, Twitter, and Reddit	Misinformation	21
Rafferty et al (2021) [117]	Content analysis		Twitter	Misinformation	12
Albucker and Lipner (2023) [118]	Cross-sectional analysis		TikTok	Misinformation	17
Tang and Park (2017) [119]	Quantitative content analysis		Pinterest	Misinformation	19
Merten et al (2020) [120]	Directed content analysis		Pinterest	Misinformation	20
Tamminga and Lipoff (2021) [121]	Mixed-methods analysis	United States	Blogs	Misinformation	20
Merten et al (2022) [122]	Exploratory study		Pinterest	Misinformation	21
Petukhova et al (2020) [123]	Retrospective review		Facebook	Misinformation	19
