# Supplementary material for: The New Media Landscape and Its Effects on Skin Cancer Diagnostics, Prognostics, and Prevention: Scoping Review
Source: JMIR Dermatol. 2024 Apr 8;7:e53373. doi: 10.2196/53373 (PMC11036192; doi:10.2196/53373)
Supplement: Multimedia Appendix 5 [file derma_v7i1e53373_app5.pdf]

			DISCERN	JAMA	PEMAT-U	PEMAT-A	GQS
Ruppert et al (2017) [98]	Evaluated Youtube videos on skin protection and skin cancer prevention	Revealed low quality videos without sustainable information, poor reliability	—	—	—	—	—
Reinhardt et al (2023) [105]	Identified and evaluated videos on skin cancer screenings on YouTube	Mediocre understandability, mediocre quality and actionability, low reliability	3.1	37.17%	64.27%	58.22%	3.72
Reinhardt et al (2022) [104]	Evaluation of videos regarding cSCC on YouTube	Revealed low to mediocre quality, videos lacked clear information sources	—	41.1%	55%	55%	3.4
Steeb et al (2022) [107]	Identified and evaluated videos about BCC on YouTube	Videos were of mediocre quality and good understanding but had low reliability and actionability	3.3	27.74%	70.8%	45.9%	—
Huang et al (2021) [100]	Review of YouTube videos regarding Mohs micrographic surgery	Poor reliability, usability, and quality	2.52	33.2%	—	—	—
Iglesias Puzas et al (2021) [101]	Reviewed YouTube video content regarding Mohs surgery	YouTube cannot be considered a reliable source, lack of information sources	1.54	—	—	—	—
Mamo et al (2021) [103]	Accessed the accuracy, quality, and reliability of THC oil and skin cancer information on YouTube	All videos included were misleading, of poor quality and reliability and poor content, comments were favorable	0	—	—	—	1
Guzman et al (2020) [99]	Assessed the quality of content based on treatment as it relates to skin cancer	Video quality is mediocre	2.3	—	—	—	—
Steeb et al (2020) [106]	Identified YouTube videos on melanoma and rated their quality, reliability, usability, and understandability	Mediocre quality, mediocre understandability, low reliability and low actionability most deductions due to the lack of references or visual aids	3.22	32.4%	82.7%	38.52%	—
Joly-Chevrier et al (2023) [102]	Assessment of skin cancer content on YouTube	Videos were low in quality and lacked validated sources	Non-med: 2.4 Medical: 3.1	Non-med: 38% Medical: 44%	—	—	—
